# Impairment of visual acuity and retinal morphology following resolved chronic central serous chorioretinopathy

**DOI:** 10.1186/s12886-019-1171-5

**Published:** 2019-07-25

**Authors:** Maciej Gawęcki, Agnieszka Jaszczuk-Maciejewska, Anna Jurska-Jaśko, Małgorzata Kneba, Andrzej Grzybowski

**Affiliations:** 1 Dobry Wzrok Ophthalmological Clinic, Gdańsk, Poland; 2 Department of Ophthalmology, Univeristy of Warmia and Mazury, Olsztyn, Poland

**Keywords:** Chronic central serous chorioretinopathy, Micropulse laser, Central retinal thickness, Subretinal fluid

## Abstract

**Purpose:**

Central serous chorioretinopathy (CSCR) is a complex ocular entity that, in its chronic form, can lead to serious visual impairment and morphological damage to the retina.

The aim of the current retrospective study was to evaluate the damage present after long-standing but resolved central serous chorioretinopathy and refer it to healthy individuals. Correlations between measurable factors—for example, duration of the disease, baseline retinal morphological parameters, or patient age and/or their degree of impairment—were also assessed.

**Materials and methods:**

The study group consisted of thirty-two eyes (13 female and 19 male, mean age 49.6 years SD +/− 10.5) with chronic central serous chorioretinopathy (mean duration 18.9 months SD +/− 15.4) in which complete resolution of subretinal fluid was achieved after subthreshold micropulse laser treatment. Inclusion criterion was a lack of subretinal fluid within the whole area of the central retina scanned by the spectral domain optical coherence tomography. The group was extracted out of 51 cases of chronic CSCR that were treated with that method. They were analyzed according to final best-corrected visual acuity and retinal morphological parameters as measured by spectral optical coherence tomography with angiography option (OCTA). Results were compared with the outcomes of a control group, which consisted of 40 eyes of healthy individuals with full distance visual acuity (0.0 logMAR, 1.0 Snellen) never treated with subthreshold micropulse laser. Statistical analysis included regarding correlation between final visual acuity and final central retinal thickness and retinal and functional parameters prior to treatment.

**Results:**

Final best-corrected visual acuity after chronic central serous chorioretinopathy was 0.23 logMAR (0.6 Snellen) and central retinal thickness was 39.32 μm smaller than in controls. No correlation was found between final visual acuity and retinal thickness and duration of the disease, patient age, and baseline morphological retinal parameters. OCTA scans revealed impaired choriocapillaries flow signal even following resolution of the disease.

**Conclusion:**

Chronic central serous chorioretinopathy is a potentially damaging clinical entity that results in serious visual impairment, retinal thinning, and choroidal flow defects. Further research is needed to determine precisely the timepoint of this damage.

## Introduction

Central serous chorioretinopathy (CSCR) is a well-known ophthalmological entity; however, its pathogenesis remains still unclear. The disease affects mainly young and active people aged between 20 years and 50 years, with strong dominance of males [1]. It has also been recognized as accompanying systemic diseases or treatments [2–4] Risk factors listed most often include stress, type A personality, high testosterone serum levels in men, hyperopia and systemic vascular diseases such as hypertension or high heart rate [5–9]. At present, most authors agree that the pathology is localized in the choroid rather than in the retinal pigment epithelium (RPE) [10–12]. Alterations of the RPE are thought to be secondary to choroidal defects or dysfunctions [13, 14].

CSCR occurs in two basic forms: acute and chronic. The acute form morphologically presents predominantly as a serous fluid under the neurosensory retina. The chronic form, on the other hand, may present in a few different ways—specifically, either as subretinal fluid (SRF) similarly to in the acute form, as a RPE detachment, or as a mixture of both. Acute CSCR usually recedes spontaneously within a period of one month to three months without any significant visual impairment. Conversely, the chronic type (chronic CSCR; CCSCR) lasts longer, even for a few years. Chronic accumulation of SRF, if central, leads to retinal thinning and the loss of photoreceptors. Patients usually end up with some degree of visual impairment, which in some cases can be significant. As the disease affects mainly young and active people, it has important socioeconomic impact. Patients have problems with such everyday activities as driving or working on a computer. Difficulties are often escalated due to the personality type A presented by the patients. For years, different therapies for CCSCR have been tested. One of the recent advances in treating CSCR is subthreshold micropulse laser therapy (SMPLT), which is effective in terms of resolution of SRF; however, in CCSCR, this treatment elicits only a moderate improvement of visual acuity [15–19]. Furthermore, the complete resolution of SRF after many months or years of active disease does not necessary mean a restoration of normal vision or normal retinal morphology will occur. To our knowledge, this damage has not yet been precisely assessed in the medical literature; thus, our study sought to evaluate functional and morphological impairments in the retina in long-standing but resolved CCSCR following SMPLT. We also considered measurable factors that could influence the amount of the damage, such as the duration of the disease, baseline retinal morphological parameters, or the age of the patient.

## Materials and methods

All procedures performed in the study were done in accordance with the ethical standards of the institutional research committee and with the principles of the 1964 Declaration of Helsinki. The study was retrospective and approved by the research committee of Dobry Wzrok Ophthalmological Clinic. The material for the study consisted of 32 eyes with CCSCR that responded well to SMPLT. For the purpose of the study only the cases in which complete resolution of SRF were chosen: subretinal fluid was absent in any part of the central retina scanned by spectral domain optical coherence tomography (SD OCT). Those 32 cases were extracted out of the cohort of 51 patients with CCSCR treated with SMPLT within the period of two years between January of 2015 and January of 2017. SMPLT treatment was performed with Supra 577 multispot yellow laser (Quantel Medical 2013). In 28 cases just one session of SMPLT treatment was needed to achieve a complete resolution of SRF. Remaining 4 cases needed 2 sessions separated by 3 months interval to achieve that goal. The study included symptomatic patients that had been under observation in our and other clinics for a period of time longer than 4 months. This meets the criteria of clinical chronicity of CSCR, which are accepted in medical literature. The average duration of symptoms in our study group was close to 19 months. Duration of the disease was assessed by reviewing patients’ medical history.

There were 19 males and 13 females in the study group. Other demographics and characteristics of the study group together with the control group are presented in Table 1.

**Table 1:** Characteristics of the study group following SMPLT and the control group. Statistical significance of the difference between the groups in age (Mann–Whitney U test), BCVA (Wilcoxon matched-pairs test) and retinal parameters (t-test)

Parameter	Study group (*n* = 32)		Control group (*n* = 40)		Significance of difference between parameters
	Mean	SD	Mean	SD	p
Age	49.56	10.48	53.80	11.15	0.14
Duration of CSCR (months)	18.91	15.42			
CRT (μm)	225.19	33.80	264.50	21.93	0.00
CV (mm^3^)	10.18	0.64	10.24	0.47	0.67
CRTA (μm)	282.84	17.96	284.13	12.74	0.72
BCVA logMAR	0.23	0.18	0.00	0.00	0.00

*CRT* central retinal thickness, *CV* cube volume, *CRTA* average central retinal thickness, *SRF* subretinal fluid, *BCVA* best-corrected visual acuity, *SD* standard deviation

The study included patients that were not previously treated by any invasive methods, such as laser photocoagulation or photodynamic therapy. They were just a subject to observation or were treated by oral or topical nonsteroid anti-inflammatory drugs without any success in other clinics. CSCR was diagnosed if the presence of SRF was noted. Sometimes SRF was accompanied by pigment epithelial detachment. Choroidal neovascularization (CNV) was excluded in all the cases. CSCR was diagnosed by the following imagining techniques: spectral domain optical coherence tomography (Zeiss Cirrus OCT with AngioPlex; Carl Zeiss Meditec AG, Jena, Germany), fluorescein angiography (FA), and fundus autofluorescence (FAF) (Zeiss FF-450; Carl Zeiss Meditec AG, Jena, Germany). The presence of SRF was determined by SD OCT and FA. FAF helped reveal alterations of the RPE typical for CCSCR: lipofuscin accumulation and loss of RPE cells. CNV was identified by FA, and patients suspected of or identified as having CNV were excluded from the study. Full ophthalmological examination was also carried out for each of the participants of the study. That included a review of their medical history and measurements of best-corrected visual acuity (BCVA) on a Snellen chart. BCVA measurements were converted according to the Logarithm of the Minimum Angle of Resolution (logMAR) scale for the purpose of statistical analysis. By the means of the SD- OCT the following parameters were determined: central retinal thickness (CRT), central retinal volume (cube volume; CV), average CRT (CRTA), and maximum SRF height. Retinal measurements using the Zeiss Cirrus SD-OCT machine (Carl Zeiss Meditec AG, Jena, Germany) were recorded in the retinal areas according to the Early Treatment Diabetic Retinopathy Study (ETDRS) grid; for these measurements, CRT refers to retinal thickness in the central circle of the posterior pole measuring 1 mm in diameter, the CRTA parameter provides the average retinal thickness within the central circle measuring 6 mm in diameter, and the CV reflects the retinal volume under the central circle measuring 6 mm in diameter. In this study we included patients with central location of SRF, however sometimes the areas of SRF involved large areas of central retina, beyond the foveal part. In this cases not only CRT parameter but also CRTA and CV could be affected.

SRF was present under the foveola in all cases; in some eyes however, its maximum accumulation was not exactly at the center of the macula. Therefore, maximum SRF height was measured and used as an additional parameter for determining the change in retinal morphology after SMPLT.

The schedule for BCVA and SD-OCT measurements was prior to treatment and at two months after each instance of SMPLT. If residual SRF was still present at two months after the first SMPLT, another laser session was scheduled for one month later (hence, a three-month interval occurred between the SMPLT sessions in these cases). At two months after the second SMPLT session, BCVA and SD-OCT measurements were then taken once again. Analysis of the measurements obtained from the study group was performed after a maximum of two laser sessions. SMPLT procedure was carried out basing on the SD-OCT retinal maps. The whole area of the SRF presence was covered with confluent foci of micropulse yellow laser (577 nm). The following laser parameters were used: focus diameter - 160 μm, the power – fixed level of 250 mW, the time of exposure - 0.2 s, and the duty cycle - 5%.

In 18 cases with resolved fluid, OCT angiography (OCTA) was performed. An analysis was completed at the level of the choriocapillaries, as follows: scan: 6 mm × 6 mm; slice: 175; and reference and offset: top RPE is + 29 μm and bottom RPE is + 49 μm.

The results of the study group were compared with OCT and BCVA parameters measured in the control group, which consisted of 40 eyes of healthy individuals (18 males and 22 females) with no present or previous ophthalmic disorders and who presented with full BCVA (0.0 logMAR). Control group consisted of individuals never treated by the SMPLT. In all cases in the control group, OCTA was performed.

There was no statistically significant difference between age and gender between the study group and the control group (chi-squared test was used for gender, Mann–Whitney U test was used for age).

The characteristics of the control group are presented in Table 1.

Additionally, we aimed to find a correlation between final BCVA and final CRT and certain parameters such as patient age, duration of disease, and baseline morphological parameters of the retina. Statistical methods for this evaluation are described in the following section.

### Statistical analysis

The statistical significance of the results of SMPLT treatment (change in functional and morphological parameters) was assessed by use of the Wilcoxon matched-pairs test.

The measure of the functional impairment after long-standing disease was the difference in BCVA between the study group and the control group. Morphological impairment was measured by the difference in retinal parameters (i.e., CRT, CRTA, and CV) between the study group and the control group.

For the difference in BCVA between the study and control group, the Mann–Whitney U nonparametric test was used. The difference in morphological parameters (i.e., CRT, CRTA, and CV) was assessed by use of a t-test for independent variables.

Correlation between final BCVA and baseline patient characteristics (e.g., age, duration of CCSCR, CRT, CRTA, CV, and SRF height) was evaluated using the Spearman rank correlation coefficient. The same test was also used to assess the correlation between final CRT and baseline patient characteristics.

Statistical analysis was performed using Statistica 10.0 (StatSoft Inc., Tulsa, OK, USA), and the following primary parameters of descriptive statistics were selected: arithmetic mean (M), median (Me), standard deviation (SD), first and third quartiles (Q1 and Q3), and the minimum (Min) and maximum (Max) values. The results were quantified as been statistically significant in cases in which the calculated probability satisfied the inequality test, or when *p* < 0.05.

## Results

The results of SMPLT treatment are presented in Table 2. The improvement of BCVA and the reduction of retinal morphological parameters were statistically significant. BCVA changed from 0.34 logMAR to 0.23 logMAR, which is equivalent to approximately a one-line improvement on the Snellen chart.

**Table 2:** Study group before and after SMPLT treatment. Statistical significance of change in BCVA and morphological parameters (Wilcoxon matched-pairs test)

Parameter	Before SMPLT			After SMPLT			Statistical significance of change in parameters
	Mean	Median	SD	Mean	Median	SD	p
CRT (μm)	338.19	308.00	111.14	225.19	228.00	33.80	0.000001
SRF height (μm)	156.84	133.50	99.09	0.00	0.00	0.00	0.000001
CV (mm^3^)	10.92	10.60	1.20	10.18	10.10	0.64	0.000004
CRTA (μm)	303.94	294.00	33.58	282.84	282.50	17.96	0.000002
BCVA logMAR	0.34	0.30	0.21	0.23	0.20	0.18	0.000146

*SMPLT* subthreshold micropulse laser treatment, *CRT* central retinal thickness, *CV* cube volume, *CRTA* average central retinal thickness, *SRF* subretinal fluid, *BCVA* best-corrected visual acuity, *SD* standard deviation

The final BCVA in the study group was 0.23 logMAR (about 0.6 Snellen). The difference between the study group and control group, in which all patients had a BCVA of 0.0 logMAR (1.0 Snellen), was significant (Mann–Whitney U test: Z = 7.827417, *p* = 0.000000).

In the study group, the retina was distinctly thinner in its central part: CRT was significantly lower in patients after CSCR as compared with in the control group. However, such a difference was not noted in the two other parameters of CV and average CRT (Table 1).

Also, baseline morphological retinal parameters (before treatment), such as CRT or retinal volume, did not correlate with the final visual acuity – Table 3.

**Table 3:** Correlation between final BCVA (logMAR) and baseline patient characteristics (e.g., age, duration of CCSCR, CRT, CRTA, CV, and SRF height before treatment) (Spearman rank correlation coefficient)

Parameter	N	R Spearman	t (N-2)	p
Age	32	0.22	1.21	0.2370
Duration of CSCR	32	0.35	2.02	0.0525
CRT	32	−0.23	−1.30	0.2022
SRF height	32	−0.22	−1.24	0.2235
CV	32	−0.07	− 0.38	0.7051
CRTA	32	−0.09	−0.52	0.6073

*CRT* central retinal thickness, *CV* cube volume, *CRTA* average central retinal thickness, *SRF* subretinal fluid

There was also a lack of correlation between final CRT and patient age, duration of the disease, and baseline visual acuity - Table 4. CRT and the height of SRF before SMPLT treatment did not correlate with final CRT either. However, a correlation was found between baseline CV and baseline average CRT and final CRT. Notably, patients with higher CV and CRTA parameters prior to SMPLT also had higher CRT values after the therapy. It must be recalled, however, that CV and CRTA parameters measure the retina according to large areas and volumes, not just the foveal part, the integrity of which is crucial for preserving visual acuity.

**Table 4:** Correlation between final retinal thickness and baseline patient characteristics (e.g., age, duration of CCSCR, CRT, CRTA, CV, and SRF height before treatment) (Spearman rank correlation coefficient)

Baseline parameter	N	Spearman	t (N-2)	p
Age	32	− 0.17	− 0.94	0.3537
Duration of CSCR	32	−0.27	−1.51	0.1415
CRT	32	0.33	1.89	0.0684
SRF height	32	0.28	1.58	0.1243
CV	32	0.39	2.34	0.0261
CRTA	32	0.45	2.74	0.0103
BCVA logMAR	32	−0.15	−0.82	0.4174

*CRT* central retinal thickness, *CV* cube volume, *CRTA* average central retinal thickness, *SRF* subretinal fluid, *BCVA* best-corrected visual acuity

### OCTA assessment

**Fig. 1:**
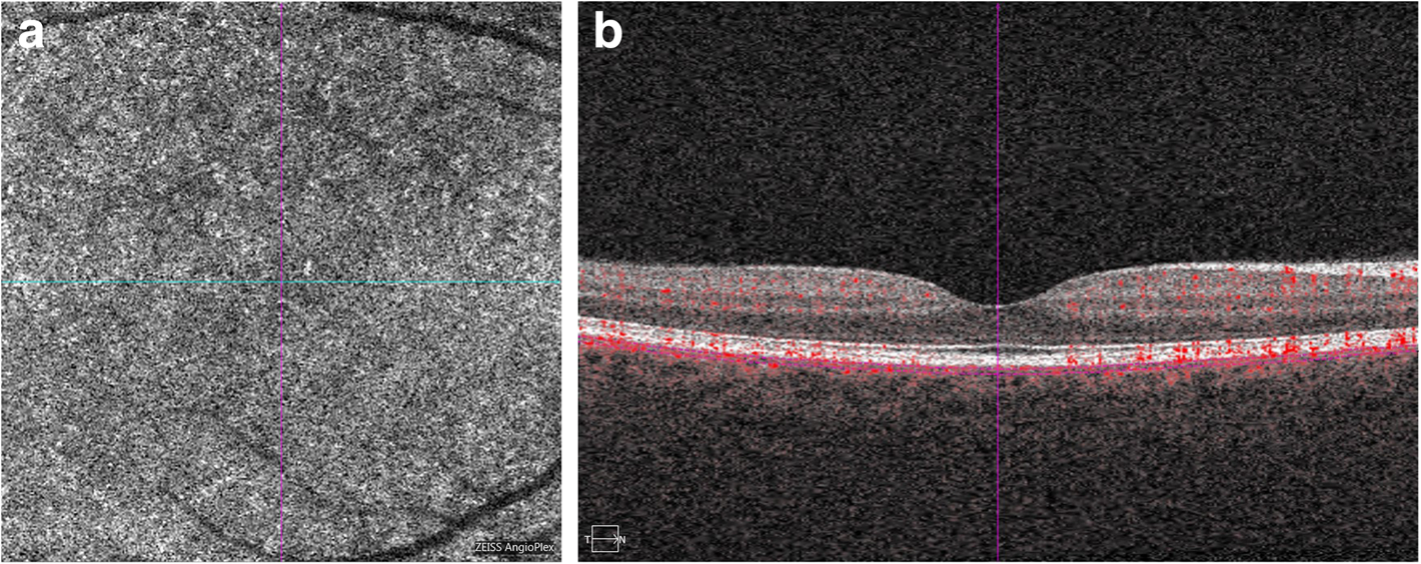
Normal image of choriocapillaries (**a**) and SD-OCT scan of the patient from the control group (**b**)

OCTA angiograms recorded at the level of the choriocapillaries were obtained from the 18 patients from the study group and compared with OCTA angiograms from the control group. Normal OCTA showed an evenly distributed “salt-and-pepper” pattern at the level of the choriocapillaries, without any vasculature defects (i.e., areas of signal void) (Fig. 1).

**Fig. 2:**
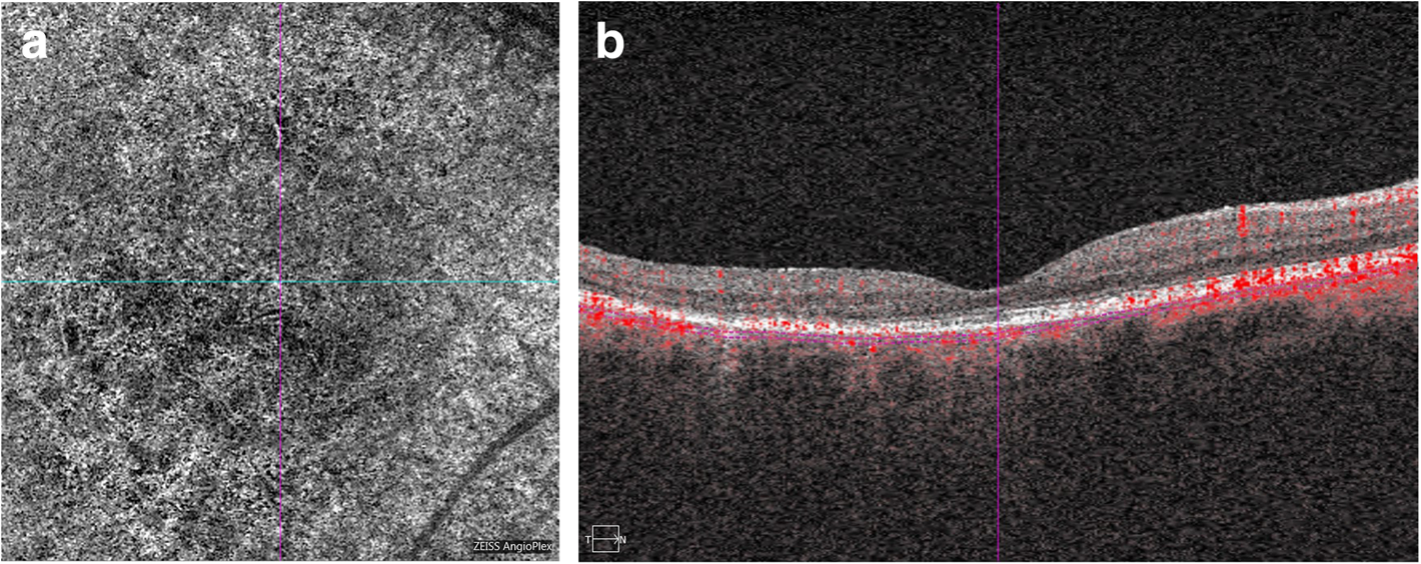
OCTA scan on the choriocapillaries level of the patient from the study group (**a**). Image reveals areas of hyporeflectance that refer to impaired choroidal vasculature. SD-OCT scan (**b**) shows retinal thinning in the central part

In all cases of the study group, a variable number of spots of hyporeflectance were noted (Fig. 2).

**Fig. 3:**
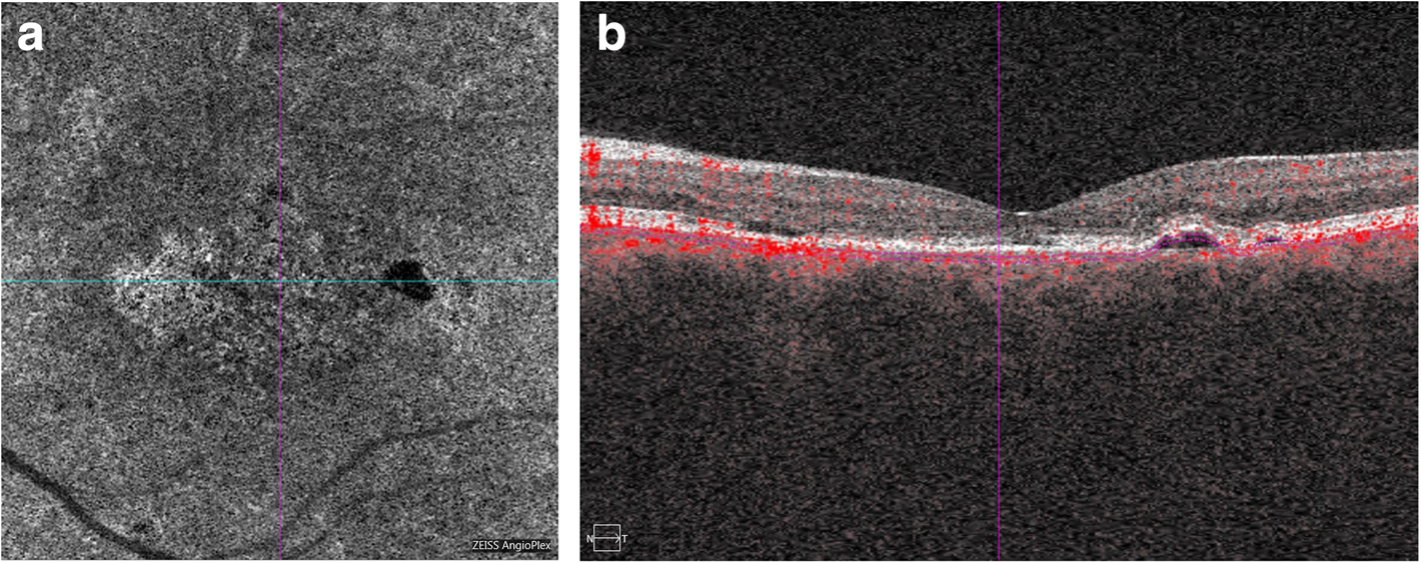
Advanced retinal thinning after resorption of subretinal fluid in chronic CSCR. SD-OCT scan (**b**) reveals retinal thinning and loss of RPE and disruption of EZ on the left side of the fovea. On the right side of the fovea scan shows small area of PED. OCTA image (**a**) presents distinct areas of hyperreflectance that respond to the areas of the loss of RPE. There are small hyporeflectannt spots in the center that refer to the areas of hypoperfusion. Area of PED on the right is visible as larger area of hyporeflectance on the OCTA scan

In some cases, especially when the retina was significantly thinner, hyporeflectant spots were accompanied by areas of hyperreflectance (Fig. 3).

Areas or spots of hyporeflectance represent areas of disturbed perfusion at the level of the chioriocapillaries. The effect of hyperreflectance are probably attributed to the “window defect” and represent better visualization of the choroidal vessels due to retinal atrophy but not due to increased blood flow.

## Discussion

Our study sought to reveal the amount of functional and morphological damage present after long-standing CSCR. Thus far, the medical literature on the subject concentrates predominantly on discussing the effects of treatment of CSCR. [15–19] Previous authors have suggested varying amounts of reduction of retinal edema and improvement in BCVA; however, the final results usually include cases with insufficient responses to SMPLT—that is, patients with persisting SRF—as well. Moreover, final CRT and BCVA values were not referred to the measurements in healthy individuals. Our study analyzed patients after complete resolution of their CCSCR and correlated its findings with the characteristics of healthy individuals. In this manner, our study provides a clear picture of visual acuity deficit and retinal thinning after CCSCR.

The exclusion of a large group of patients with completely resorbed SRF after CCSCR might be a problem. This disease has a long course with remissions and recurrences, so it is difficult to find a point at which reliable measurements can be performed. In our study, we chose a group that responded well to SMPLT. As patients were followed up with every two months, it was possible to identify any points of remission and to obtain BCVA and OCT measurements.

Other studies that have employed SMPLT in the treatment of CSR usually have concentrated on an improvement in BCVA and the reduction of CRT [15, 17–22]. Final BCVA is usually given as an average value of the whole cohort of patients, which is composed of both responders and nonresponders.

Koss et al. reported a final BCVA of 51.6 ± seven letters on the ETDRS grid (equivalent to 0.7 logMAR), [23] while Malik et al. reported a final BCVA of 45.5 ± 12 letters (0.8 logMAR), [19] Scholz et al. reported a final BCVA of 0.3 ± 0.25 logMAR, [16] and Kim et al. reported a final BCVA of 0.04 ± 0.06 logMAR [18]. However, we have to remember that these scores might be downplayed, as they include nonresponders with poorer BCVA values.

In our study, final BCVA after chronic CSCR was significantly reduced to 0.23 ± 0.18 logMAR (0.6 Snellen), which signals a loss of approximately four lines of Snellen visual acuity. This visual defect, if unilateral, can be compared with moderate anisometropic amblyopia. Affected patients may have peripheral stereopsis, which enables normal everyday activity; however, more demanding visual tasks have to be conducted with the use of the unaffected eye.

BCVA in the study group improved by 0,11logMAR only and this outcome remains in consent with the results of other studies [16, 18, 19, 23]. Scholz et al. in their meta-analysis of results of SMPLT in CCSCR from 14 studies (216 patients) report similar mean BCVA improvement of 6.34 ETDRS letters (range − 15 to 20) [24]. Relatively poor rate of visual improvement in CCSCR is also noted with other treatment modalities. BCVA gain in the treatment of chronic CSCR with photodynamic therapy (PDT) is similar to SMPLT, usually at the level of a few ETDRS letters or 1 Snellen line [25–27]. Few studies however, show larger amount of BCVA improvement after PDT [28, 29]. Those inconsistences may be a consequence of different characteristics of the study groups, especially in regard to the duration of the disease and baseline visual acuity. Studies directly comparing SMPLT and PDT in the treatment of chronic CSCR generally do not favor any of these therapies and reveal moderate BCVA improvement (a few ETDRS letters) similar in both groups [30–32]. Only large PLACE trial shows significantly better morphological results with PDT treatment and moderately better visual outcome compared to SMPLT (+ 6.78 ETDRS letters versus + 4.48 ETDRS letters at 7 months). Vision related quality of life, however was the same in both groups [33].

Therapy with mineralocorticoid inhibitors (eplerenone) if effective at all, brings similar functional benefits as SMPLT and PDT. Meta-analysis of 5 randomized control trials evaluating efficacy of eplerenone treatment in CCSCR revealed just 0.1logMAR difference in BCVA between patients treated and receiving placebo at two months [34]. Most of the studies of that subject show moderate BCVA improvement, usually by less or about 0,1logMAR [35–37].

Laser photocoagulation is of limited use in chronic CSCR as it requires the leakage point to be located outside the fovea. In CCSCR usually we approach a variety and multiplicity of symptoms that include subretinal fluid, pigment epithelial detachment and alterations of the retinal pigment epithelium, often without well demarcated leakage points. In performed clinical trials, laser photocoagulation in CSCR did not influence visual outcome in the long term [38]. Moderate BCVA improvement can be achieved in selected cases with navigated laser photocoagulation [39].

Most of the studies report mean retinal thickness values after SMPLT that include also patients with retinal edema, so the numbers are potentially higher and do not reflect actual retinal thinning.

We found that the central retina in the study group measured 225.19 ± 33.8 μm in the foveal part, which was on average 39.32 μm thinner than the same areas in the controls. Retinal thinning in the fovea after resorption of SRF is less if the retina is generally thicker in the macular area prior to treatment (i.e., if the patient has higher CRTA and CV values); in other words, there exists a positive correlation. Retinal thinning in chronic CSCR has been confirmed by other studies; for example, Breukink et al. reported progressive retinal thinning of 15.1 μm per year in patients with chronic presence of SRF. However, these authors did not present final CRT values [40].

Interestingly, in our study, parts of the central retina outside the fovea were not that significantly affected. Average thickness and volume values of the central part of the retina did not vary significantly from the measurements taken in healthy individuals. Probably, the presence of SRF affects mainly the thinnest and very central part of the retina, which has only one source of blood supply: the choroid. As we already know, it is the choroid that is the primary location of the lesion that underlies the pathogenesis of serous chorioretinopathy. Disturbance of choroidal perfusion in this area affects also the outer retina and photoreceptors, at which point, loss of the RPE and photoreceptors subsequently occurs, which explains the functional damage.

Our OCTA findings seem to confirm our hypothesis. Our patients showed impaired choroidal perfusion at the level of the choriocapillaries after long-term chorioretinopathy despite remission of the disease.

The existing medical literature confirms choroidal flow disturbances in active CSCR. Rochepeau et al. and Cakir et al. analyzed choroidal perfusion in CSCR and revealed the existence of decreased blood flow at the level of chioriocapillaries in the active stage of the disease [41, 42]. Perfusion improved with the resorption of SRF. Matet et al. presented similar results [43]; disturbances of choroidal perfusion in their study increased with patient age, duration of CSCR, and disease severity. The choriocapillaries showed thinning and larger choroidal vessel dilation at the affected sites. Decreased density of the choriocapillaries in CSCR was also noted by Cardillo et al. [44].

An analysis of our findings proves that chronic cases of CSCR present with choroidal flow disturbances even after the absolute cessation of symptoms. All of the resolved cases of chronic CSCR in our study showed an impairment of the flow signal at the level of the choriocapillaries. Focusing on the resolved cases is a novel protocol in comparison with in other studies, which largely covered defects of choroidal flow in OCTA of patients with the active form of the disease [45].

Choroidal vasculature alterations are rarely reported in the literature after other treatments in CCSCR. Toto et al. did not find any changes in retinal capillary plexuses or choroidal vasculature after eplerenone treatment of CSCR [46]. Fujita et al. presented a small series of 6 patients in whom OCTA revealed choriocapillaries recovery after half-dose PDT treatment (improvement of flow signal) [47]. Similar effect was observed by Xu et al. [48]. Definitely more research is needed to more precisely assess vascular changes in the course of treatment of CSCR. That will certainly happen with development of OCTA angiography technique and software.

The present study did not find any correlation between patient age, duration of symptoms, or baseline retinal morphology and final visual acuity after remission of symptoms. Also, final central retinal thickness, a morphological parameter that was significantly affected by the disease (retinal thinning), did not correlate with the duration of the disease.

These findings are in agreement with those of our previous study [49]. Theoretically, a longer duration of the disease should result in larger degrees of functional and morphological damage. If this does not happen, however, than it is possible that major functional and morphological defects occur within the first months of the active disease when no treatment is applied according to medical recommendations [50, 51]. As such, the logical conclusion would be to recommend prompt treatment of cases of CSCR already in the acute form, especially if we do not know whether the acute disease will become chronic or not. Nevertheless, at this point this suggestion has to be treated as a pure hypothesis and needs to be confirmed in further studies including control group with CSCR that is just observed. To our knowledge, the only study so far that directly analyzed the effects of SMPLT in acute CSCR versus observation was published in 2018. Arora et al. showed that patients with acute CSCR in whom prompt SMPLT treatment was administered had better final BCVA and contrast sensitivity in comparison with patients with acute CSCR who were observed only [52].

Definitely, more research is needed to provide new recommendations for the treatment of both acute CSCR and chronic CSCR. It would be useful to more precisely indicate the position of SMPLT and photodynamic therapy (PDT) in the treatment of this disease, as these two methods are the most promising and seem to provide the best functional and morphological results [53–55].

## Conclusion

CCSCR appears to be a potentially serious ophthalmological disease that can lead to visual impairment. After long-standing disease, final BCVA is decreased and the retina is significantly thinner in the central part. Furthermore, choroidal perfusion is impaired even following the remission of symptoms. Lack of correlation between the duration of the disease and amount of visual deficit suggests, that the disease does not progress in a linear way. Major damage might occur within the first months of the disease, that’s why more research is needed to support prompt treatment of CSCR without waiting for spontaneous remission, as it has been recommended in previous years.

## Data Availability

The datasets used and/or analyzed during the current study are available from the corresponding author on reasonable request. Dobry Wzrok Ophthalmological Clinic e-mail: rejestracja@dobry-wzrok.pl.

## Abbreviations

BCVA: best corrected visual acuity
CCSCR: chronic central serous chorioretinopathy
CNV: choroidal neovascularization
CRT: central retinal thickness
CRTA: central retinal thickness average
CSCR: central serous chorioretinopathy
CV: cube volume
ETDRS: Early Treatment Diabetic Retinopathy Study
FA: fluorescein angiography
FAF: fundus autofluorescence
OCTA: optical coherence tomography angiography
PDT: photodynamic therapy
RPE: retinal pigment epithelium
SD: standard deviation
SD-OCT: spectral domain optical coherence tomography
SMPLT: subthreshold micropulse laser treatment
SRF: subretinal fluid
